# Epidemiology, tumour characteristics, treatment and outcomes associated with spinal nerve sheath tumours: a systematic review protocol

**DOI:** 10.1136/bmjopen-2023-083011

**Published:** 2024-10-11

**Authors:** Omar Ali Mahdi, Maria Gharios, Adnan Fatfat, Victor Gabriel El-Hajj, Aman Singh, Erik Edström, Adrian Elmi-Terander

**Affiliations:** 1 Department of Clinical Neuroscience, Karolinska Institutet, Stockholm, Sweden; 2 Stockholm Spine Center Capio, Löwenströmska Hospital, Stockholm, Sweden; 3 Department of Medical Sciences, Örebro University, Örebro, Sweden

**Keywords:** Systematic Review, Neurology, NEUROSURGERY

## Abstract

**Abstract:**

**Introduction:**

Nerve sheath tumours arise from both the central and peripheral nervous systems. In particular, cases of spinal or paraspinal origins are scarce and poorly covered in the literature. This systematic review aims to summarise the body of evidence regarding spinal nerve sheath tumours and assess its quality, to provide the current knowledge on epidemiology, tumour characteristics, diagnostics, treatment strategies and outcomes.

**Methods and analysis:**

Databases including PubMed, Web of Science and Embase will be searched using keywords such as “spinal”, “nerve sheath”, “neurofibroma”, “schwannoma”, “neurinoma” and “neurilemoma”. The search will be limited to studies published no earlier than 2000 without language restrictions. Case reports, editorials, letters and reviews will be excluded. Reference lists of identified studies will be searched to find possible additional relevant records. Identified studies will be screened for inclusion, by one reviewer at first and then two independent ones in the next step to increase the external validity. The Rayyan platform will be used for the screening and inclusion process. Data extraction within several predetermined areas of interest will proceed. Subjects of interest include epidemiology, histopathology, radiological diagnostics, surgery, complications, non-surgical treatment alternatives, disease outcomes and predictors of outcome, and recurrence rates. On satisfactory amount of homogenous data, a meta-analysis of key outcomes such as recurrence risk or postoperative neurological improvement will be performed. This systematic review will primarily serve as a reference guide to aid in diagnosis and treatment of patients with spinal schwannomas, while also spotlighting the knowledge gaps in the literature to help guide future research initiatives.

**Ethics and dissemination:**

Ethics approval is not required for the protocol or review as both are based on existing publications. For dissemination, the final manuscript will be submitted to a peer-reviewed journal.

**STRENGTHS AND LIMITATIONS OF THIS STUDY**

- A strategy was designed to thoroughly assess both evidence levels and risk of bias of individual studies.
- A broad search strategy and limited exclusion criteria allow for a larger number of studies to be identified, which then permits a multi-perspective coverage of the topic.
- The quality of data may not suffice to perform a proper quantitative statistical analysis which limits the level of evidence that can be achieved. However, on satisfactory data quality, a meta-analysis will be performed.

## Introduction

 Spinal nerve sheath tumours are typically benign intradural extramedullary (IDEM) nerve sheath tumours often originating from the dorsal root of spinal nerves. While some studies indicate that these tumours are the most common IDEM tumours, others disagree, in favour of spinal meningiomas.^1^,^4^ Apart from being IDEM, spinal or paraspinal schwannomas can be located either exclusively extradurally or both intradurally and extradurally, where they are sometimes referred to as dumbbell tumours.^5^ Regardless, the incidence of spinal schwannomas, the most common subtype among nerve sheath tumours, is reported to range between 0.3 and 0.4 cases/100 000/per year.^6 7^ Typically, there is no sex predisposition, and the peak age at diagnosis is between 40 and 70 years.^1 8^

Schwannomas, also referred to as neurilemomas or neurinomas, are mainly classified as WHO grade I,^1 9^ while malignant schwannomas, let alone spinal ones, are scarce and typically present with larger tumour sizes (usually larger than 5 cm) and a high tendency to recur.^7^ On the molecular level, schwannomas arise from sporadic and solitary uncontrolled growth of Schwann cells with loss of myelin production.^1 10^ Nonetheless, cases with multiple tumours do exist and are commonly associated with one of two syndromes: neurofibromatosis type 2 (NF2) or schwannomatosis. In fact, approximately 1% of spinal schwannomas^11^ and 4% of sporadic ones^12 13^ have been linked to NF2, a genetic condition resulting from the loss of function in the merlin (also known as schwannomin) coding NF2 tumour suppressor gene on chromosome 22.^12^,^15^ Both familial (autosomal dominant) and sporadic cases of NF2 have been documented.^12 13^ On the other hand, schwannomatosis, the rarest type of neurofibromatosis,^16^ has reportedly been associated to 2%–4.6% of schwannoma cases.^17 18^ Most commonly, schwannomatosis is associated with a germline mutation in the tumour suppressor genes SMARCB1 or LZTR1.^16 19 20^ The loss of genetic material in both NF2 and schwannomatosis can either arise sporadically or as part of a familial condition.^21 22^

Histologically, schwannomas typically feature two architectural patterns: Antoni A and Antoni B. While the former features compact elongated cells with normochromic nuclei and nuclear palisading, the latter exhibits hypocellularity, with less compact cells, ovoid and smaller nuclei, and more lipidization. The vasculature of the schwannomas is composed of hyalinised thick-walled vessels.^10 14^ Other benign nerve sheath tumours include neurofibromas; however, unlike schwannomas, these tumours typically have smaller nuclei and lack hyalinisation of vasculature.^10^

Although well encapsulated and lacking metastatic potential, benign spinal schwannomas are capable of local expansion and neurovascular compromise. This may afflict a great amount of discomfort and distress in patients, who generally present with severe radiculopathies and other symptoms such as lower back pain, motor, sensory or sphincter dysfunction, depending on the location of the tumour.^619 23^,^25^ On presentation, the diagnosis is usually established with the help of MRI. These tumours classically display a heterogenous T2 hyperintensity, where the areas of higher signal intensity typically correlate with intratumoral cystic degeneration and hypointensity to isointensity on T1 imaging. Irregular gadolinium contrast filling and rim enhancement of the spinal lesion may also be present.^3 26 27^

The gold standard of treatment for spinal schwannomas is gross total resection (GTR) with focus on the preservation of neurovascular structures. In fact, GTR of benign non-infiltrative tumours has been repeatedly shown to decrease the risk of recurrence.^28 29^ Resection is typically achieved through posterior approach with laminectomy or laminotomy and subsequent dural opening.^30^,^33^ Due to the risk of postoperative spinal instability, a haemilaminectomy may be preferred and advocated by some. Anterior and anterolateral approaches may be reserved for tumours located in the subaxial spine. For dumbbell schwannomas located at the cervical level, the surgical strategy may be determined by the Toyama classification which categorises these tumours into nine different groups with respect to the anatomical relation to neighbouring structures like the dura and the intervertebral foramen.^34^ It is worth mentioning that the resection of dumbbell tumours of the spine may be accompanied by several technical and anatomical challenges, which may often limit the maximal achievable grade of resection.

Although the use of intraoperative neuromonitoring (IONM) is often reserved for intramedullary spinal cord tumours, this technology has already paved the way into spinal schwannoma surgeries, under claims of related significant postoperative improvements.^6 30^

Outcomes of spinal schwannoma surgery are generally favourable with postoperative neurological improvements witnessed in most patients.^6^ Viereck *et al* even concluded a significant improvement in quality of life after operation of spinal schwannomas.^35^ Additionally, perioperative complications are rare, which makes the procedure both efficacious and safe. In fact, a study conducted by Halvorsen *et al* demonstrated the 1-year and 5-year postoperative survival rates to be around 98% and 95%, respectively.^9^ Operative complications associated with spinal schwannoma resections range from neurological deterioration to cerebrospinal fluid leaks and wound infections.^13^

If surgery is performed and after resection of the tumours, immunohistochemistry may be useful in the diagnostic workup of schwannomas, as S100, Ki67, P53 and CD57 have proven valuable as prognostic markers in the disease. In fact, it was shown that S100-negativity, Ki67>5%, P53-positivity and CD57-negativity all carry poorer prognosis, as the presence or absence of these markers may indicate a higher malignancy risk, a higher tendency to recurrence or poorer survival outcomes.^7^

Tumour recurrence or regrowth is a major postoperative concern, encountered in 4% to 9% of cases operated with a GTR^5 11^ and up to 30% for cases operated with a subtotal resection (STR).^36^ For patients with tumour recurrence or in cases where surgery is contraindicated, available therapeutic alternatives are radiotherapy and stereotactic radiosurgery.^30 37^ Tissue fibrosis from previous surgeries is a concern, since recurrent tumours may present with a more adherent growth pattern, making surgical interventions more complex. In their study, Kufeld *et al* could conclude that radiosurgery for spinal schwannomas was a safe and effective treatment option.^37^

This intended systematic review aims to outline the existing state-of-the-art alluding to spinal schwannomas, by going through epidemiology, diagnostics, treatment options, tumour characteristics, neurological outcomes, quality of life, mortality and recurrence, in order to serve as the basis of knowledge and improve the quality of care in patients suffering from this tumour. Moreover, the review aims at spotlighting the knowledge gap within the field in hope of stimulating and guiding future research initiatives.

## Methods and analysis

### Study registration

This protocol is in accordance with the Preferred Reporting Items for Systematic Reviews and Meta-Analyses Protocol (PRISMA-P) statement of 2015.^38^ The PRISMA-P checklist is provided as online supplemental file 1. The systematic review protocol will also be registered on PROSPERO, before submission of the final manuscript to a peer-reviewed journal.

### Eligibility criteria

#### Inclusion criteria

##### Type of studies

All peer-reviewed and original studies, published after 2000, and available in the PubMed, Embase or Web of Science databases are eligible for inclusion.

##### Type of participant

All patient groups will be considered for inclusion, regardless of age, ethnicity and sex. Similarly, all spinal nerve sheath tumours irrespective of size, tumour grading or other tumor-specific characteristics will be regarded in the analysis. A reliable histological or radiological diagnosis of the tumour must, however, be presented. Malignant nerve sheath tumours may be excluded depending on the final sample size reached.

##### Type of interventions

All diagnostic modalities and treatment methods of spinal schwannomas will be included to cover the entirety of the literature present.

##### Type of outcome measurements

Epidemiological data such as age, sex and socioeconomic factors, as well as possible predictors of poor outcomes like comorbidity and spinal cord compression, will also be addressed. Furthermore, patient outcome objects including pain, neurological function, health-related quality of life and tumour recurrence status, as well as tumor-specific characteristics such as the markers of proliferative activity and WHO grade will be included. Additional outcomes presented in the selected studies may subsequently be considered. In those cases, the possibility of reporting biases will be dealt with accordingly.

### Exclusion criteria

Reviews, editorials, letters to the editor, conference abstracts and case and technical reports will be disregarded from this review. Publications prior to the year 2000 will not be included in the review as a means to highlight the most recent works in the literature.

### Databases and search strategy

An electronic database search will be performed on PubMed, Embase and Web of Science (online supplemental file 2). The search will be broad to avoid missing valuable studies. Due to their abundance, case reports will be filtered out at that stage using the necessary tools provided by each of the databases. Appropriate filters will also be used to exclude studies published prior to the year 2000. A reference list search of the included studies as well as expert consultation will also be performed to screen for any eligible article that may have been missed.

### Study selection

The records retrieved from the different databases will be exported into Zotero,^39^ for the removal of duplicates. The clean list of records will then be screened based on title and abstract by two reviewers. In the next step, the remaining record will be assessed by full-text reading, by three independent and blinded reviewers. Studies will then be selected based on compliance with the eligibility criteria previously mentioned. This process will be entirely managed from the Rayyan platform.^40^ Potential disagreements after pooling of the results will be resolved by team discussion and subsequent unanimous decision. The operation as described will be meticulously depicted in a PRISMA flowchart which will be provided.

### Data extraction

Data extraction from the selected records will take place using a predefined Google Sheets^41^ extraction template, preliminarily including (1) general information—title, first author, journal, publication year, etc; (2) patient characteristics and epidemiology—age, sex, tumour location, grade, etc; (3) intervention characteristics—imaging, adjuvant therapy, etc; (4) study characteristics—study type, sample size, follow-up time, etc; and (5) outcomes—neurological outcomes, quality of life, recurrence rate, mortality rate, follow-up time, adverse events and their management, main conclusions, etc. The final work will be assessed and cross-checked by multiple reviewers to prevent any error and to obtain a reliable body of raw data.

### Assessment of risk of bias

The Oxford Center for Evidence-Based Medicine (OCEBM) system,^42^ modified by Wright *et al*, will be used to assess evidence levels^43 44^ (table 1). Each article will receive an evidence level ranked from 1 to 4 (I–IV), since our review and the set of eligibility criteria inherently imply the exclusion of level V studies. Afterwards, the risk of bias assessment will take place, and an individual score (IS) will be proposed as follows: using the evidence level as a starting point, studies with deemed low risk of bias will be upgraded while those with a higher risk of bias will get downgraded. Risk of bias will be assessed using the appropriate tools specific to the type of study, as defined by Ma *et al*.^45^ The final IS will range from I to IV. An identical approach has already been described in previous works.^46^,^51^

**Table 1:** Level of evidence based on primary research question by Wright *et al*^43^

Evidence levels	Therapeutic studies—investigating the results of treatment	Prognostic studies—investigating the outcome of disease	Diagnostic studies—investigating a diagnostic test
Level I	Good-quality randomised controlled trial Systematic review of level I studies	Prospective study Systematic review of level I studies	Testing of previously developed diagnostic criteria in series of consecutive patients (with universally applied reference ‘gold’ standard) Systematic review of level I studies
Level II	Prospective cohort study Poor-quality randomised controlled trial Systematic review Level II studies Nonhomogeneous level I studies	Retrospective study Study of untreated controls from a previous randomised controlled trial Systematic review of level II studies	Development of diagnostic criteria on basis of consecutive patients (with universally applied reference ‘gold’ standard) Systematic review of level II studies
Level III	Case-control study Retrospective cohort study Systematic review of level III studies		Study of non-consecutive patients (no consistently applied reference ‘gold’ standard) Systematic review of level III studies
Level IV	Case series (with no, or historical, control group)	Case series	Case-control study Poor reference standard
Level V	Expert opinion	Expert opinion	Expert opinion

### Quality of evidence of the included studies

The Grading of Recommendations Assessment, Development, and Evaluation (GRADE)^52^ approach will be used to rate the body of evidence supporting the key study outcomes in our review. This approach relies on factoring in the risk of bias assessment scores, inconsistency, indirectness, imprecision, large effect magnitude, dose-response gradient, etc to obtain a final quality of evidence grade of ‘high’, ‘moderate’, ‘low’ or ‘very low’ (table 2).^52^ Using the Guideline Development Tool (GRADEpro GDT),^53^ a summary of findings and/or GRADE evidence table for key study outcomes will be generated.

**Table 2:** Quality of evidence grades from the GRADE Handbook (Chapter 5)

Quality	Definition
High	We are very confident that the true effect lies close to that of the estimate of the effect.
Moderate	We are moderately confident in the effect estimate; the true effect is likely to be close to the estimated effect, but it may be substantially different.
Low	Our confidence in the effect estimate is limited; the true effect may be substantially different from the estimate of the effect.
Very low	We have very little confidence in the effect estimate; the true effect is likely to be substantially different from the estimate of effect.

### Data synthesis and meta-analysis

After extraction and appraisal, the data will be systematically presented in the context of a narrative and qualitative synthesis. Topics of interest to this review are chosen as follows:

1. Epidemiology,
2. Histology,
3. Imaging and diagnostics,
4. Surgical treatment and technique,
5. Complications, avoidance and management,
6. Alternative non-surgical options and adjuvant treatment,
7. Neurological outcomes and quality of life,
8. Recurrence.

For sections where a quantitative risk analysis is in question, a meta-analysis will be employed on the condition that a satisfactory amount of data, not limited by severe methodological heterogeneity issues, is secured. If these conditions are not met, we will proceed with a narrative synthesis of data as originally planned.

### Statistical analysis

In the event of a meta-analysis, assessment of publication bias—using funnel plots and Egger’s test—as well as for heterogeneity—using the Q and I^2^ tests—will be performed as per the recommendations.^54^ On high heterogeneity, forest plots based on random effect models will be used. All statistical analyses will be performed using R packages.^55^

### Patient and public involvement

Patients were not involved in the design or conception of the study.

## Ethics and dissemination

Ethics approval is not required for this systematic review as it is based on publications that are available to the public. Dissemination of this work will be achieved through submission to a peer-reviewed journal where the results will be openly available.

## Discussion

The objective of this systematic review is to outline the current knowledge regarding spinal nerve sheath tumours in the literature and thereby create an overview to assist physicians in diagnosis and treatment of the disease. The present publications concerning spinal nerve sheath tumours cover multiple elements including incidence,^1 6 7 11^ age and sex distrubution,^7 15 56^ location of tumour,^11 12 24 25 30 56^ treatment and outcomes.^2 24 31 57 58^ Most of the molecular and cellular knowledge on schwannomas of the spine originates from studies performed on non-spinal schwannomas. Moreover, despite the fact that a plethora of studies agree on several fundamental concepts regarding spinal schwannomas, there are still a lot of contradictory and imprecise data concerning topics such as sex propensity,^1 8^ location of the tumour,^11 24 30 56^ its prevalence among other spinal tumours^1^,^4^ and recurrence rates.^5 11^ Other limitations inherent to the literature found concerns the short follow-up times,^7 11 37^ the retrospective character of studies^7 11 13 19 33 57^ and the small sample sizes present.^3 5 25 31 36 57^ The contradictions identified across the literature, along with the paucity of studies and the weak evidence base provided by individual studies, call for a gathering and pooling of the data to achieve more precise information with increased external validity.^59^

Due to heterogeneity of both study design and outcomes, a meta-analysis may be difficult to carry out; however, in case a satisfactory amount of homogenous data is uncovered, meta-analysis will be sought for key outcomes, such as neurological and functional outcomes, recurrence rates and mortality.

Finally, the intended PRISMA-compliant review aims at becoming a reliable source of aggregated references, synthesised in a logical and systematic manner, to convey the state-of-the-art in the field. Further knowledge and increased accessibility of the topic will ultimately enhance the understanding of spinal schwannomas and their treatment. Furthermore, this systematic review will also benefit and facilitate future research by highlighting and acknowledging the areas in the literature on spinal schwannomas where gaps in knowledge may exist.^54^ To the best of our knowledge, the absence of systematic reviews focused on spinal schwannomas currently makes this review a forerunner in its domain.

## supplementary material

10.1136/bmjopen-2023-083011 online supplemental file 1

10.1136/bmjopen-2023-083011 online supplemental file 2

## References

[1] Tish S, Habboub G, Lang M (2020). The epidemiology of spinal schwannoma in the United States between 2006 and 2014. J Neurosurg Spine.

[2] Ando K, Kobayashi K, Nakashima H (2020). Surgical outcomes and factors related to postoperative motor and sensory deficits in resection for 244 cases of spinal schwannoma. J Clin Neurosci.

[3] Ando K, Imagama S, Ito Z (2016). How do spinal schwannomas progress? The natural progression of spinal schwannomas on MRI. J Neurosurg Spine.

[4] Ogasawara C, Philbrick BD, Adamson DC (2021). Meningioma: A Review of Epidemiology, Pathology, Diagnosis, Treatment, and Future Directions. Biomedicines.

[5] Emel E, Abdallah A, Sofuoglu OE (2017). Long-term Surgical Outcomes of Spinal Schwannomas: Retrospective Analysis of 49 Consecutive Cases. Turk Neurosurg.

[6] Hohenberger C, Hinterleitner J, Schmidt N-O (2020). Neurological outcome after resection of spinal schwannoma. Clin Neurol Neurosurg.

[7] Li B, Li J, Miao W (2018). Prognostic Analysis of Clinical and Immunohistochemical Factors for Patients with Spinal Schwannoma. World Neurosurg.

[8] Jinnai T, Koyama T (2005). Clinical characteristics of spinal nerve sheath tumors: analysis of 149 cases. Neurosurgery.

[9] Halvorsen CM, Rønning P, Hald J (2015). The Long-term Outcome After Resection of Intraspinal Nerve Sheath Tumors: Report of 131 Consecutive Cases. Neurosurgery.

[10] Antonescu CR, Louis DN, Hunter S, Louis DN, Ohgaki H, Wiestler OD (2016). WHO classification of tumours of the central nervous system.

[11] Parlak A, Oppong MD, Jabbarli R (2022). Do Tumour Size, Type and Localisation Affect Resection Rate in Patients with Spinal Schwannoma?. Med Bogota Colomb.

[12] Safaee M, Parsa AT, Barbaro NM (2015). Association of tumor location, extent of resection, and neurofibromatosis status with clinical outcomes for 221 spinal nerve sheath tumors. Neurosurg Focus.

[13] Safaee MM, Lyon R, Barbaro NM (2017). Neurological outcomes and surgical complications in 221 spinal nerve sheath tumors. J Neurosurg Spine.

[14] Belakhoua SM, Rodriguez FJ (2021). Diagnostic Pathology of Tumors of Peripheral Nerve. Neurosurgery.

[15] Palmisciano P, Ferini G, Watanabe G (2022). Surgical Management of Craniovertebral Junction Schwannomas: A Systematic Review. *Curr Oncol*.

[16] Chick G, Victor J, Poujade T (2018). Sporadic Schwannomatosis: A Systematic Review Following the 2005 Consensus Statement. J Neurol Surg A Cent Eur Neurosurg.

[17] Huang JH, Simon SL, Nagpal S (2004). Management of patients with schwannomatosis: report of six cases and review of the literature. Surg Neurol.

[18] Antinheimo J, Sankila R, Carpén O (2000). Population-based analysis of sporadic and type 2 neurofibromatosis-associated meningiomas and schwannomas. Neurology (ECronicon).

[19] Li P, Zhao F, Zhang J (2016). Clinical features of spinal schwannomas in 65 patients with schwannomatosis compared with 831 with solitary schwannomas and 102 with neurofibromatosis Type 2: a retrospective study at a single institution. J Neurosurg Spine.

[20] Tamura R (2021). Current Understanding of Neurofibromatosis Type 1, 2, and Schwannomatosis. Int J Mol Sci.

[21] Evans DGR (2009). Neurofibromatosis type 2 (NF2): A clinical and molecular review. Orphanet J Rare Dis.

[22] Gonzalvo A, Fowler A, Cook RJ (2011). Schwannomatosis, sporadic schwannomatosis, and familial schwannomatosis: a surgical series with long-term follow-up. Clinical article. *J Neurosurg*.

[23] Ottenhausen M, Ntoulias G, Bodhinayake I (2019). Intradural spinal tumors in adults-update on management and outcome. Neurosurg Rev.

[24] Mualem W, Ghaith A-K, Rush D (2022). Surgical management of sacral schwannomas: a 21-year mayo clinic experience and comparative literature analysis. *J Neurooncol*.

[25] Jeon JH, Hwang HS, Jeong JH (2008). Spinal schwannoma; analysis of 40 cases. J Korean Neurosurg Soc.

[26] De Verdelhan O, Haegelen C, Carsin-Nicol B (2005). MR imaging features of spinal schwannomas and meningiomas. J Neuroradiol.

[27] Liu WC, Choi G, Lee S-H (2009). Radiological findings of spinal schwannomas and meningiomas: focus on discrimination of two disease entities. Eur Radiol.

[28] Newman WC, Berry-Candelario J, Villavieja J (2021). Improvement in Quality of Life Following Surgical Resection of Benign Intradural Extramedullary Tumors: A Prospective Evaluation of Patient-Reported Outcomes. Neurosurgery.

[29] Safavi-Abbasi S, Senoglu M, Theodore N (2008). Microsurgical management of spinal schwannomas: evaluation of 128 cases. J Neurosurg Spine.

[30] Apostolov G, Kitov B, Poryazova E (2021). Sporadic spinal schwannomas and neurofibromas - a review. Folia Med (Plovdiv).

[31] Lee SE, Jahng TA, Kim HJ (2015). Different Surgical Approaches for Spinal Schwannoma: A Single Surgeon’s Experience with 49 Consecutive Cases. World Neurosurg.

[32] Sun I, Pamir MN (2017). Non-Syndromic Spinal Schwannomas: A Novel Classification. Front Neurol.

[33] Lenzi J, Anichini G, Landi A (2017). Spinal Nerves Schwannomas: Experience on 367 Cases-Historic Overview on How Clinical, Radiological, and Surgical Practices Have Changed over a Course of 60 Years. Neurol Res Int.

[34] Ito K, Aoyama T, Miyaoka Y (2015). Surgical Strategies for Cervical Spinal Neurinomas. Neurol Med Chir (Tokyo).

[35] Viereck MJ, Ghobrial GM, Beygi S (2016). Improved patient quality of life following intradural extramedullary spinal tumor resection. J Neurosurg Spine.

[36] Sohn S, Chung CK, Park SH (2013). The fate of spinal schwannomas following subtotal resection: a retrospective multicenter study by the Korea spinal oncology research group. J Neurooncol.

[37] Kufeld M, Wowra B, Muacevic A (2012). Radiosurgery of spinal meningiomas and schwannomas. Technol Cancer Res Treat.

[38] Moher D, Shamseer L, Clarke M (2016). Preferred reporting items for systematic review and meta-analysis protocols (PRISMA-P) 2015 statement. Rev Esp Nutr Hum Diet.

[39] (2016). Roy Rosenzweig center for history and new media. Computer software.

[40] Ouzzani M, Hammady H, Fedorowicz Z (2016). Rayyan-a web and mobile app for systematic reviews. Syst Rev.

[41] Google sheets: online spreadsheet editor | Google workspace. https://www.google.com/sheets/about/.

[42] OCEBM Levels of Evidence Working Group* "The oxford 2011 levels of evidence”. Oxford centre for evidence-based medicine. http://www.cebm.net/index.aspx?o=5653.

[43] Wright JG, Swiontkowski MF, Heckman JD (2003). Introducing levels of evidence to the journal. J Bone Joint Surg Am.

[44] Anderson KK, Tetreault L, Shamji MF (2015). Optimal Timing of Surgical Decompression for Acute Traumatic Central Cord Syndrome: A Systematic Review of the Literature. Neurosurgery.

[45] Ma LL, Wang YY, Yang ZH (2020). Methodological quality (risk of bias) assessment tools for primary and secondary medical studies: What are they and which is better. Mil Med Res.

[46] El-Hajj VG, Stenimahitis V, Gharios M (2023). Spontaneous spinal cord infarctions: a systematic review and pooled analysis protocol. BMJ Open.

[47] El-Hajj VG, Pettersson Segerlind J, Burström G (2022). Current knowledge on spinal meningiomas: a systematic review protocol. BMJ Open.

[48] Tatter C, El-Hajj VG, Fletcher-Sandersjöö A (2023). Radiographic measurements for the prediction of dysphagia after occipitocervical fusion: a systematic review. Acta Neurochir (Wien).

[49] El-Hajj VG, Pettersson-Segerlind J, Fletcher-Sandersjöö A (2022). Current Knowledge on Spinal Meningiomas Epidemiology, Tumor Characteristics and Non-Surgical Treatment Options: A Systematic Review and Pooled Analysis (Part 1). Cancers (Basel).

[50] El-Hajj VG, Pettersson-Segerlind J, Fletcher-Sandersjöö A (2022). Current Knowledge on Spinal Meningiomas-Surgical Treatment, Complications, and Outcomes: A Systematic Review and Meta-Analysis (Part 2). Cancers (Basel).

[51] Iop A, El-Hajj VG, Gharios M (2022). Extended Reality in Neurosurgical Education: A Systematic Review. *Sensors (Basel*).

[52] Schünemann H, Brożek J, Guyatt G (2013). GRADE Handbook for Grading Quality of Evidence and Strength of Recommendations.

[53] (2020). GRADEpro GDT: gradepro guideline development tool (developed by Evidence Prime, Inc.). McMaster University.

[54] Lasserson TJ, Thomas J, Higgins JPT, Higgins JPT, Thomas J, Chandler J (2021). Cochrane Handbook for Systematic Reviews of Interventions version 6.2.

[55] R Core Team (2020). R: a language and environment for statistical computing. https://www.R-project.org.

[56] Parsa AT, Lee J, Parney IF (2004). Spinal cord and intradural-extraparenchymal spinal tumors: current best care practices and strategies. J Neurooncol.

[57] Sowash M, Barzilai O, Kahn S (2017). Clinical outcomes following resection of giant spinal schwannomas: a case series of 32 patients. SPI.

[58] Ng Z, Ng S, Nga V (2018). Intradural Spinal Tumors-Review of Postoperative Outcomes Comparing Intramedullary and Extramedullary Tumors from a Single Institution’s Experience. World Neurosurg.

[59] Edwards TB (2014). What is the value of a systematic review?. J Shoulder Elbow Surg.

