# Supplementary material for: Epidemiology, tumour characteristics, treatment and outcomes associated with spinal nerve sheath tumours: a systematic review protocol
Source: BMJ Open. 2024 Oct 11;14(10):e083011. doi: 10.1136/bmjopen-2023-083011 (PMC11481236; doi:10.1136/bmjopen-2023-083011)
Supplement: online supplemental file 2 [file bmjopen-14-10-s002.docx]

**Preliminary search strategy on the different databases:**

**PubMed:** Spin* AND (schwannoma OR neurilem* OR neurinoma) NOT case report Filters: Humans, from 2000 - 2024/01/01

**Embase:** spin* AND (schwannoma OR neurilem* OR neurinoma) AND [humans]/lim AND [english]/lim AND [2000-2023]/py NOT 'case report' AND ([article]/lim OR [article in press]/lim OR [data papers]/lim OR [erratum]/lim OR [preprint]/lim)

**Web Of Science:** (((ALL=("spinal schwannoma") AND PY=(2000-2500) AND LA=(English) NOT TS=("CASE REPORT")) NOT (TASCA==("VETERINARY SCIENCES") OR DT==("EDITORIAL MATERIAL" OR "LETTER" OR "MEETING ABSTRACT"))) OR ((TI=(spinal AND schwannoma*) AND PY=(2000-2500) AND LA=(English) NOT TI=(case report)) NOT (TASCA==("VETERINARY SCIENCES") OR DT==("EDITORIAL MATERIAL" OR "LETTER" OR "MEETING ABSTRACT")))) AND ALL=(spin*)
