# Supplementary material for: Epidemiology, tumour characteristics, treatment and outcomes associated with spinal nerve sheath tumours: a systematic review protocol
Source: BMJ Open. 2024 Oct 11;14(10):e083011. doi: 10.1136/bmjopen-2023-083011 (PMC11481236; doi:10.1136/bmjopen-2023-083011)
Supplement: Supplementary file 3 [file bmjopen-2023-083011.reviewer_comments.pdf]

## PEER REVIEW HISTORY

BMJ Open publishes all reviews undertaken for accepted manuscripts. Reviewers are asked to complete a checklist review form and are provided with free text boxes to elaborate on their assessment. These free text comments are reproduced below.

### ARTICLE DETAILS

**Title (Provisional)**

Epidemiology, tumor characteristics, treatment, and outcomes associated with spinal nerve sheath tumors: a systematic review protocol

**Authors**

Mahdi, Omar Ali; Gharios, Maria; Fatfat, Adnan; El-Hajj, Victor Gabriel; Singh, Aman; Edström, Erik; Elmi-Terander, Adrian

---

### VERSION 1 - REVIEW

---

| | |
|---|---|
| **Reviewer** | **1** |
| **Name** | **Yousefi, Omid** |
| **Affiliation** | **Shiraz University of Medical Sciences** |
| **Date** | **18-Feb-2024** |
| **COI** | **None** |

---

As a protocole for a systematic review, this work is well written and has provided enough of what is going to take place.

---

| | |
|---|---|
| **Reviewer** | **2** |
| **Name** | **Metikala, Sreenivasulu** |
| **Affiliation** | **VCU, Orthopaedic Surgery** |
| **Date** | **06-Apr-2024** |
| **COI** | **None** |

---

Well designed protocol. I agree to its publication.

---

### VERSION 1 - AUTHOR RESPONSE

Thank you for the reviewers comment. We appreciate the positive feedback and are looking forward to get this manuscript disseminated.

Reviewer: 3

Thank you. Simpson grade has now been removed.

---

## VERSION 2 - AUTHOR RESPONSE

This was now fixed
